# An integral method for determining the molecular composition of lignin and its application

**DOI:** 10.1038/s41598-022-23884-5

**Published:** 2022-11-09

**Authors:** Qingzhi Ma, Xuejin Zhang

**Affiliations:** Key Laboratory of Recycling and Eco-Treatment of Waste Biomass of Zhejiang Province, School of Environmental and Nature Resources, Zhejiang University of Science and Technology, Hangzhou, 310023 Zhejiang China

**Keywords:** Method development, Analytical chemistry

## Abstract

Lignin is a natural and renewable aromatic polymer, but only about 2% of lignin is utilized with high added value. Polydispersity and heterogeneity are the key reasons for the difficulty in separation, fractionation, characterization, purification and utilization of lignin. However, the molecular weight of lignin is still described from the overall perspective of number-/weight-average molecular weight (Mn and Mw), which if far from enough to understand the heterogeneous and dispersed lignin. To provide a tool for understanding the molecular weight of lignin from a molecular perspective, an integral method for quantifying the molecular characteristics of lignin molecules at arbitrary molecular intervals on the molecular weight distribution curve of lignin was established. The molecular contents of wheat straw lignin as well as its soluble and insoluble fractions at different intervals were calculated. The ease of fractionation of small molecules with weights lower than 8000 g/mol into soluble fractions, and that of large molecules with weights higher than 10,000 g/mol into insoluble fractions were quantitatively analyzed. The established integral method will significantly help in the understanding the properties of lignin at the molecular-level, as well as the fractionation and utilization of lignin.

## Introduction

Lignin is the most abundant natural renewable aromatic polymer, and it can be used to produce biomass-based chemicals and materials, such as dispersants, adhesives, activated carbon, carbon fiber, and phenolic resin^1–6^. Therefore, the process of lignin refining can be potentially used to replace the process of petroleum refining. However, the different raw materials and separation methods, and the diversity of subunits, functional groups, and linkages, result in the high heterogeneity of lignin. The high heterogeneity of lignin and the induced performance instability of the compounds significantly increase the difficulty of its high-value-added applications^7,8^. At present, lignin (except lignosulfonate) is primarily used for combustion, and its value has not been fully exploited. It is noteworthy that combustion causes the carbon captured in lignin through photosynthesis to be released back into the atmosphere in the form of carbon dioxide, which increases the load of carbon neutrality.

The controllable fractionation of heterogeneous lignin into several lignin fractions characterized by good homogeneity and stable properties is the key to realizing the high value-added applications of lignin^9–13^. Lignin fractionation methods primarily include the processes of membrane ultrafiltration, gradient acid precipitation, and sequential solvent extraction. The membrane ultrafiltration method fractionates lignin based on its molecular weight and particle size. However, the membrane pores are prone to clogging, resulting in a reduction in flux and interception size. It also results in a reduced polymer dispersion index (PDI) of the resulting fractions^14–16^. The gradient acid precipitation method is usually used to separate lignin from black liquor^17^. Large and small molecules of lignin are more prone to precipitation under high (about 10.0) and low pH (about 2.0) conditions, respectively. However, under high pH conditions, some small molecular lignin precipitates together with large molecular lignin, resulting in a high PDI. It is difficult to completely precipitate lignin of small molecules under low pH conditions. This results in low separation efficiency. The sequential solvent extraction method is used to fractionate lignin into one insoluble and two or more soluble fractions based on the solubility of lignin in one or more solvents^9,18,19^. The sequential solvent extraction method is also one of the most promising methods that can be used to obtain homogenous lignin. The operating costs, in this case, are not as high as the operation costs of the membrane method. Moreover, the degree of pollution generated under these conditions is not as high as the degree of pollution generated using the gradient acid precipitation method^8^. However, the controllable fractionation of lignin with high polydispersity has not been achieved.

The molecular weight of lignin is closely related to its physico-chemical properties^20–23^. The higher the molecular weight of lignin: (1) the smaller the exposure probability of phenolic hydroxyl, alcohol hydroxyl, and carboxyl groups and the lower their contents^24^, and (2) the higher the glass transition temperature (T_g_) and the thermal degradation temperature. Furthermore, the zeta potential, hydrophile–lipophile balance value, antioxidant, and antibacterial properties of lignin also varied with the molecular weight of lignin^20–23^. The fractionation degree of lignin molecular weight largely influences the physical and chemical properties of fractions. However, the lignin fractionation characteristic of molecular weight is usually described by the number-average molecular weight (Mn), weight-average molecular weight (Mw), and PDI. The Mn, Mw, and PDI values represent the overall molecular characteristics of lignin. However, these do not reflect the differences between the lignin molecules belonging to different molecular intervals. Therefore, lignin fractionation characteristics should be understood at the molecular level. Gel permeation chromatography (GPC), a subset of size-exclusion chromatography (SEC), is the most popular method to determine the relative weight-/number- average molecular weight and molecular weight distribution of lignin^25–28^. Polystyrene is used as the calibration to measure the molecular weight of lignin because of their similar structure. Even though, the hydrodynamic volume of polystyrene not exactly the same as acetylated lignin, the molecular weight obtained by GPC largely reflects the real results and widely accepted^25–28^. Light scattering (such as static light scattering) is considered to be the most effective method for determining the absolute weigh- molecular weight of lignin^29–32^. However, the molecular weight distribution of lignin cannot be directly obtained by light scattering method. The light scattering detector was usually connected to the end of hromatographic column (like the column used in GPC system) to obtain the molecular weight distribution message of lignin^29,31^. For GPC analysis, the lignin should be acetylated and dissolved in tetrahydrofuran (THF) which may have some influence on the molecular weight results. Overall consideration, GPC method was the most common method to test the number-average molecular weight (Mn), weight-average molecular weight (Mw), polymer dispersion index, and molecular weight distribution of lignin.

In this study, an integral method to calculate the molecular composition of lignin and the fractionation yields of the molecules belonging to arbitrary molecular intervals based on the molecular weight distribution curve of lignin was derived. Two lignin samples were fractionated into soluble and insoluble fractions, then the fractionation characteristics of lignin molecules in different intervals (two. three, and eight) were analyzed. This study will help us understand the lignin fractionation behavior from a molecular point of view and provide key support for the actualization of controllable fractionation. This will subsequently help in the high-value utilization of lignin.

## Materials and methods

### Lignin samples

Wheat straw lignin containing guaiacyl (G), syringyl (S), and *p*-hydroxyphenyl (H) units were prepared and used for fractionation^33^. Briefly, wheat straw was cooked using the formic acid (FA)–acetic acid (AA)–water (35:50:20, w/w/w) system in the solid-to-liquor ratio of 1:6 (w/w) at 140 °C over a period of 30 min. Post cooking, the mixture was filtrated to obtain the black liquor. The black liquor was concentrated and then dropped into a large amount of water to precipitate lignin. Finally, the precipitated lignin was collected by filtration, washed three times with deionized (DI) water, and freeze-dried to obtain wheat straw organic acid lignin (WL). The use of plants in the present study complies with institutional guidelines.

Masson pine lignin containing G units was also prepared for fractionation. Masson pine was cooked at the effective alkali charge of 22% and the sulfidity of 20% (both effective alkali and sulfidity are as Na_2_O), and the solid-to-liquor ratio of 1: 4.5. The cooking temperature was increased from ambient temperature to 165 °C in 90 min. The sample was held at this temperature for 60 min for the reaction. After the reaction, the mason pine black liquor was obtained by filtrating the mixture. Subsequently, the liquid was concentrated to obtain total solids of approximately 40%. The concentrated black liquor was heated to 80 °C, following which the pH was adjusted to 2.0 using 20% H_2_SO_4_ to precipitate lignin. The mixture was cooled in an ice–water bath overnight to fully precipitate lignin. Finally, the precipitated lignin was collected following the process of filtration, washed three times with DI water, and freeze-dried to obtain Masson pine kraft lignin (ML). Kraft lignin are the most important source of industrial lignin today, and ML can be a representative of kraft lignin.

### Lignin fractionation based on the process of sequential solvent extraction

To obtain soluble and insoluble lignin with the ratios of 42 ± 1%: 58 ± 1%, AA–H_2_O (6:4, v/v) and FA–AA–H_2_O (3:4:3, v/v/v) were chosen to fractionate WL and ML, respectively, based on the previously reported results obtained from the study of lignin solubility^33^. Lignin (2.5 g) was accurately weighed and placed in a 20-mL glass vial. Following this, the dissolving solvent (AA–H_2_O or FA–AA–H_2_O; 10.00 mL) and a small rotor were added to the glass vial, and the mixture was stirred at 300 rpm for 30 min at 25 °C for lignin dissolution. After dissolution, the mixture in the vial was transferred to a centrifuge tube and centrifuged at 6000 g for 10 min. The soluble fraction of lignin was centrifuged into the supernatant, and the insoluble fraction of lignin was left behind in the solid. The supernatant was carefully poured out and collected, following which it was concentrated using a rotary vacuum evaporator. The concentrated solution was washed three times by adding DI water and evaporated to completely remove formic acid and acetic acid. The last evaporated material was freeze-dried to obtain the soluble fractions (SWL and SML) of lignin.

After the supernatant was poured out, the remaining solid was collected. The insoluble solid was washed three times with DI water and centrifuged to remove the dissolving solvent. The insoluble fractions of lignin (ISWL and ISML) were finally obtained by freeze-drying the solid obtained from the final stage of centrifugation.

### Analysis of the molecular weight distribution

The lignin and fractions were acetylated using a solution of pyridine–acetic anhydride (1:1, v/v), and the protocols previously reported by us were followed^34^. The molecular distribution of the acetylated lignin was determined using the gel permeation chromatography (GPC; LC-20AD, Shimadzu, Japan) technique. A KF-804L column (Shimadzu, Japan) and a refractive index detector (RID 10A) were used for sample analysis. The flow rate was maintained at 1.0 mL/min, and the column temperature was maintained at 40 °C. Calibration was performed using eight polystyrenes of weights 162, 2400, 4050, 9880, 17,900, 27,500, 46,300, and 172,400 g/mol. According to the peak time and area of the lignin sample through the chromatographic column, the molecular weight (*Mw*) and molecular weight ratio (*Mw%*) of lignin and fractions were obtained.

## Results and discussion

### Integral method for calculating the fractionation yield of lignin belonging to a certain molecular interval

#### Lignin fractionation and molecular weight determination

When parent lignin (*L*_*0*_) was fractionated into fractions of lignin 1 (*L*_*1*_), lignin 2 (*L*_*2*_), and lignin x (*L*_*x*_), their masses were determined and set as *m*_*0*_, *m*_*1*_, *m*_*2*_, and *m*_*x*_ (g), respectively. The relationship between molecular weight and molecular weight ratio for parent lignin and the fractions were determined and set as *Mw*_*0*_ − *Mw*_*0*_*%* and *Mw*_*x*_ − *Mw*_*x*_*%*, respectively.

#### Yield of the fraction

The yield of the fraction *L*_*x*_ ($Y_{{{\text{L}}x \to total}}$), or the yields of all the molecules in *L*_*x*_, can be calculated based on Eq. (1) as follows:

1 $$ Y_{{{\text{L}}x \to total}} = \frac{{m_{x} }}{{m_{0} }}. $$

#### Lignin-mole

Based on the mass–mole calculation method [Eq. (2)], the mole of the parent lignin and the fractions can be calculated based on Eqs. (3-1) and  (3-2), respectively.

2 $$ m = n \times {\text{M}}{.} $$

Here, *m* is the mass of the pure substance in g, *n* is the mole mass (or the amount of substance) in mol, and M is the molar mass of substance in g/mol.

3-1 $$ m_{0} = \int_{\min }^{\max } {n_{0} } \times Mw_{0} \% dMw_{0} , $$

3-2 $$ m_{x} = \int_{\min }^{\max } {n_{x} } \times Mw_{x} \% dMw_{x} . $$

Here *n*_*o*_, *n*_*1*_, *n*_*2*_, and *n*_*x*_ (in mol) are the moles of the parent lignin and fractions, and min and max are the minimum and maximum molecular weights of the lignin samples, respectively.

The values of *m*_*0*_, *m*_*x*_, *Mw*_*0*_ − *Mw*_*0*_*%*, and *Mw*_*x*_ − *Mw*_*x*_*%* can be measured, and *n*_*0*_ and *n*_*x*_ can be expressed by Eq. (4-1) and  (4-2), respectively, as follows:

4-1 $$ n_{0} = \frac{{m_{0} }}{{\int_{\min }^{\max } {Mw_{0} \% } dMw_{0} }}, $$

4-2 $$ n_{x} = \frac{{m_{x} }}{{\int_{\min }^{\max } {Mw_{x} \% } dMw_{x} }}. $$

#### Lignin mass for a certain molecular interval

The masses of parent lignin and the fractions in the molecular interval of a–b ($m_{{0 \to {\text{a - b}}}}$ and $m_{{x \to {\text{a - b}}}}$) can be calculated by Eqs. (5-1) and Eq. (5-2), respectively, as follows:

5-1 $$ m_{{0 \to {\text{a}} - b}} = \int_{a}^{b} {n_{0} } \times Mw_{0} \% dMw_{0} , $$

5-2 $$ m_{x \to a - b} = \int_{a}^{b} {n_{x} } \times Mw_{x} \% dMw_{x} , $$

where a and b are the molecular weights of lignin and satisfy the relationship of min ≤ a < b ≤ max.

Equations (6-1) and (6-2) were obtained by substituting Eqs. (4-1) and (4-2) into Eqs. (5-1) and  (5-2), respectively.

6-1 $$ m_{{0 \to {\text{a}} - b}} = \frac{{\int_{a}^{b} {Mw_{0} \% } \times dMw_{0} }}{{\int_{\min }^{\max } {Mw_{0} \% } \times dMw_{0} }} \times m_{0} , $$

6-2 $$ m_{{x \to {\text{a}} - b}} = \frac{{\int_{a}^{b} {Mw_{x} \% } \times dMw_{x} }}{{\int_{\min }^{\max } {Mw_{x} \% } \times dMw_{x} }} \times m_{x} . $$

#### Molecular composition of lignin belonging to a certain molecular interval

The molecular composition (or content) of the parent lignin and the fractions in the molecular interval of a–b ($m_{{0 \to {\text{a - b}}}} (\% )$ and $m_{{x \to {\text{a - b}}}} (\% )$) can be calculated using Eqs. (7-1) and  (7-2), respectively, as follows:

7-1 $$ m_{{0 \to {\text{a}} - b}} (\% ) = \frac{{\frac{{\int_{a}^{b} {Mw_{0} \% } \times dMw_{0} }}{{\int_{\min }^{\max } {Mw_{0} \% } \times dMw_{0} }} \times m_{0} }}{{m_{0} }} , $$

7-2 $$ m_{{x \to {\text{a}} - b}} (\% ) = \frac{{\frac{{\int_{a}^{b} {Mw_{x} \% } \times dMw_{x} }}{{\int_{\min }^{\max } {Mw_{x} \% } \times dMw_{x} }} \times m_{x} }}{{m_{x} }} . $$

Two similar equations are presented below:

8-1 $$ m_{{0 \to {\text{a}} - b}} (\% ) = \frac{{\int_{a}^{b} {Mw_{0} \% } \times dMw_{0} }}{{\int_{\min }^{\max } {Mw_{0} \% } \times dMw_{0} }} , $$

8-2 $$ m_{{x \to {\text{a}} - b}} (\% ) = \frac{{\int_{a}^{b} {Mw_{x} \% } \times dMw_{x} }}{{\int_{\min }^{\max } {Mw_{x} \% } \times dMw_{x} }}. $$

#### Yield of the fraction belonging to a certain molecular interval

The yield of the fraction in the molecular interval of a–b ($Y_{Lx \to a - b}$) corresponding to the parent lignin belonging to the same interval can be calculated using Eq. (9).

9 $$ Y_{Lx \to a - b} = \frac{{m_{x \to a - b} }}{{m_{0 \to a - b} }}. $$

Equation (10) was obtained by substituting Eqs. (6-1) and (6-2) into Eq. (9).

10 $$ Y_{Lx \to a - b} = \frac{{\frac{{\int_{a}^{b} {Mw_{x} \% } d_{Mwx} }}{{\int_{\min }^{\max } {Mw_{x} \% } d_{Mwx} }}}}{{\frac{{\int_{a}^{b} {Mw_{0} \% } d_{Mw0} }}{{\int_{\min }^{\max } {Mw_{0} \% } d_{Mw0} }}}} \times Y_{Lx \to total} . $$

#### Ratio between the yield of a fraction belonging to a certain molecular interval and the yield of the fraction

The ratio ($R_{Lx \to a - b}$) between the yield of the fraction belonging to the molecular interval of a–b and the yield of the fraction can be calculated using Eq. (11).

11 $$ R_{Lx \to a - b} = \frac{{Y_{Lx \to a - b} }}{{Y_{Lx \to total} }}. $$

Equation (12) was obtained by substituting Eqs. (1) and (10) into Eq. (11).

12 $$ R_{Lx \to a - b} = \frac{{\frac{{\int_{a}^{b} {Mw_{x} \% } d_{Mwx} }}{{\int_{\min }^{\max } {Mw_{x} \% } d_{Mwx} }}}}{{\frac{{\int_{a}^{b} {Mw_{0} \% } d_{Mw0} }}{{\int_{\min }^{\max } {Mw_{0} \% } d_{Mw0} }}}}. $$

$R_{Lx \to a - b}$ can be used to evaluate the dissolution and fractionation trends of the molecules of the parent lignin between a–b:

1. If its value is greater than 1, the molecules of the parent lignin between a–b are more likely fractionated into *L*_*x*_;
2. If its value is equal to 1, the molecules of the parent lignin between a–b are equal proportionally fractionated into *L*_*x*_;
3. If its value is less than 1, the molecules of the parent lignin between a–b are difficultly fractionated into *L*_*x*_.

### Molecular weight distributions of the parent lignin and fractions

**Figure 1:**
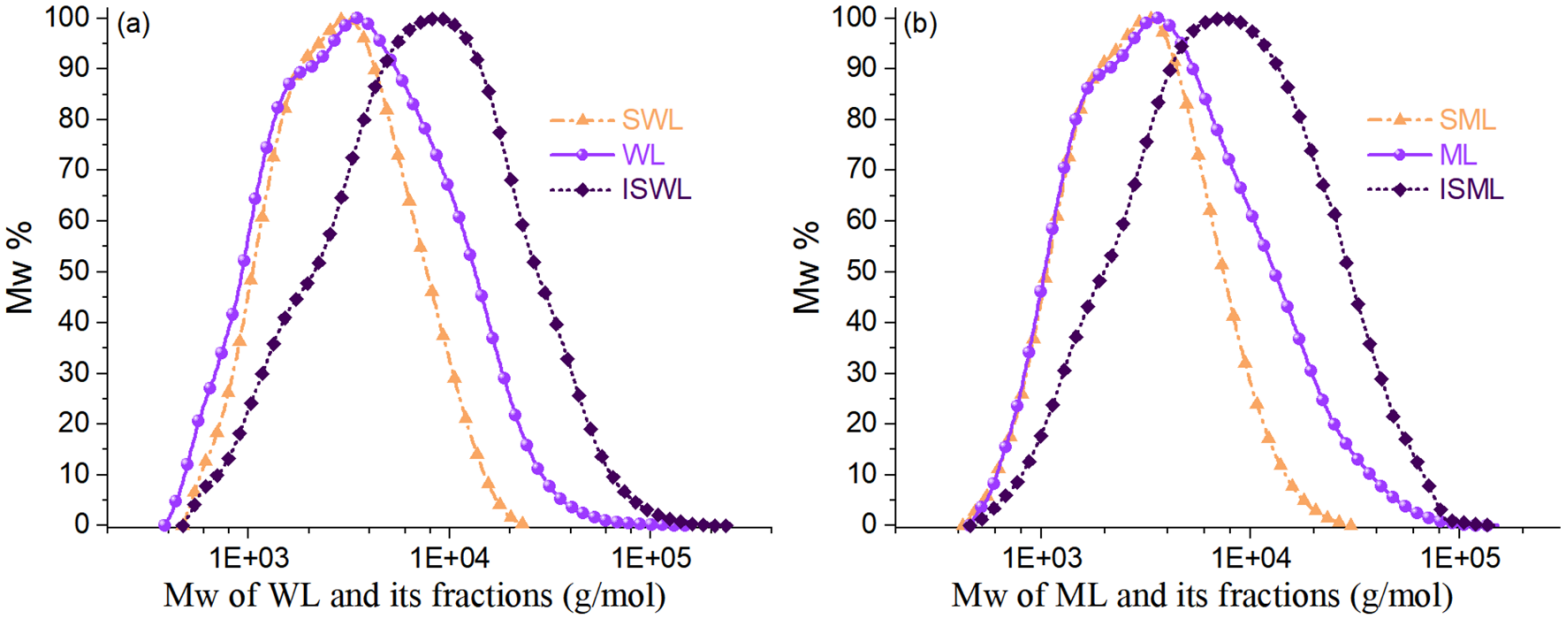
Molecular weight distribution curves generated for the lignin units and the fractions: (**a**) WL, SWL, and ISWL; (**b**) ML, SML, and ISML.

Both WL and ML were fractionated into soluble and insoluble fractions of the ratio 42 ± 1%: 58 ± 1%. The molecular weight distributions of WL, ML, and their fractions are presented in Fig. 1, and the Mn, Mw, and PDI are shown in Table 1. WL showed the Mw of 5653 g/mol. As expected, the value was higher than the Mw of SWL (3775 g/mol) and lower than the Mw of ISWL (11,975 g/mol) (Table 1). This indicates that the small molecules of lignin are more easily soluble than large ones. The PDI of SWL (1.69) was lower than the PDI of WL (2.41), indicating that the molecular distribution of SWL was narrower. This is because molecules larger than 20,000 g/mol are excluded, and only the molecules smaller than 20,000 g/mol are dissolved and fractionated into SWL (Fig. 1a). ISWL with a PDI of 2.79 presented a broader molecular distribution than WL with a PDI of 2.41 (Table 1), which consistent with the methanol fractionated fraction showed higher PDI than parent lignin^23^ and inconsistent with the gradient acid fractionated fraction showed lower PDI than parent lignin^17^. Some of the molecules, the molecular weights of which were smaller than 20,000 g/mol, and most of the molecules, the molecular weights of which were larger than 20,000 g/mol, were fractionated into ISWL (Fig. 1a), resulting in the wide distribution. ML also showed: (1) a higher Mw and broader molecular distribution than SML, and (2) a lower Mw and narrower molecular distribution than ISML (Fig. 1b). The small and large molecules of WL and ML showed different dissolution and fractionation abilities at the soluble fraction yield of 42 ± 1%. However, it is difficult to quantitatively describe the fractionation difference between small and large molecules using the parameters of Mw and PDI.

**Table 1:** Mn, Mw, and PDI corresponding to the lignin and fractions.

Lignin sample	Mn (g/mol)	Mw (g/mol)	PDI
SWL	2240	3775	1.69
WL	2346	5653	2.41
ISWL	4282	11,975	2.79
SML	2244	3770	1.68
ML	2707	6553	2.42
ISML	5021	17,791	3.54

### Fractionation characteristics of lignin molecules at different molecular intervals

**Figure 2:**
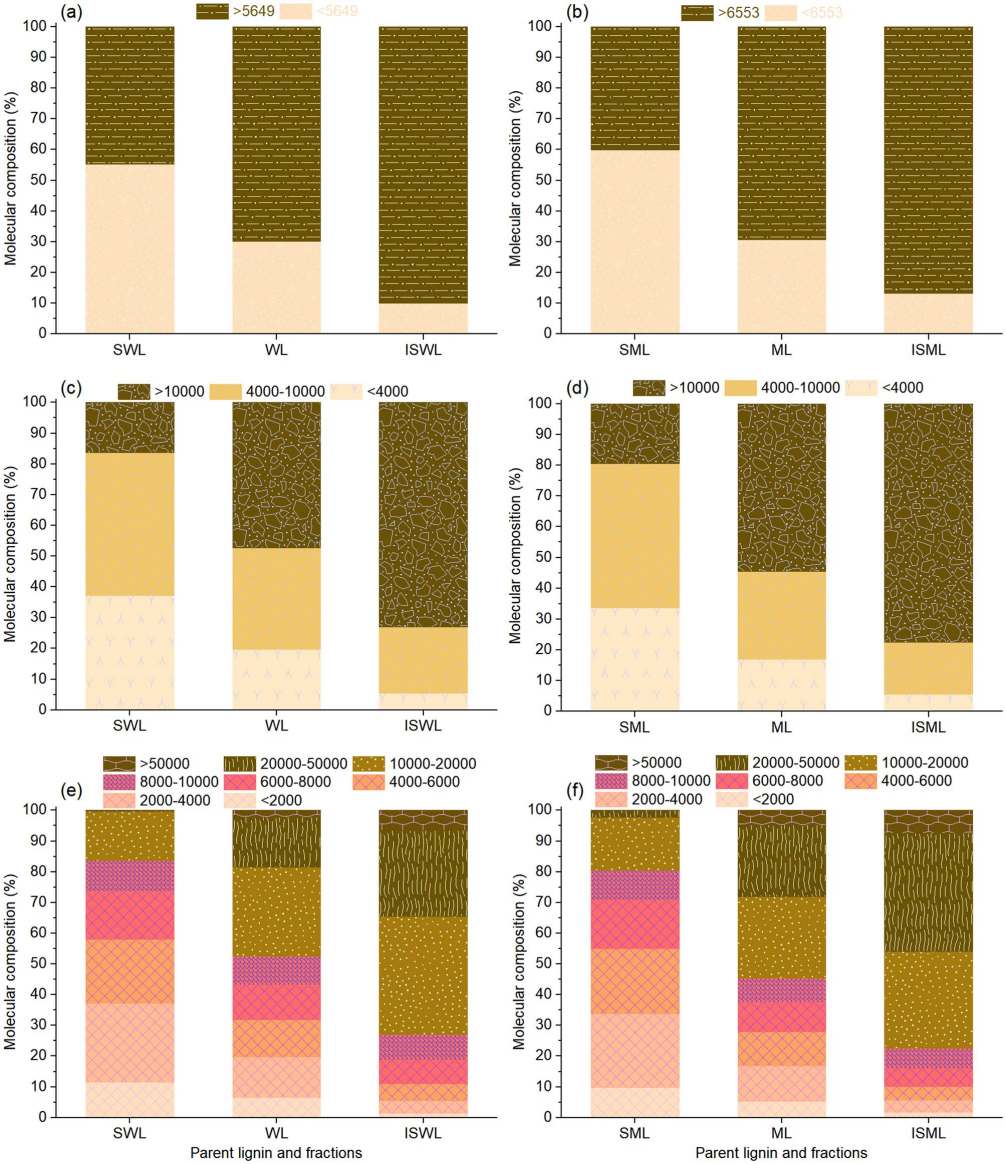
Molecular composition of the parent lignin and the fractions under different molecular intervals: WL, SWL, and ISWL under (**a**) 2, (**c**) 3, and (**e**) 8 intervals; ML, SML, and ISML under (**b**) 2, (**d**) 3, and (**f**) 8 intervals.

The fractionation characteristics of WL and ML in different molecular intervals were calculated to verify the validity of the established integral method. During the calculation process, WL and ML are seen as parent lignin (*L*_0_), SWL and SML as *L*_1_, and ISWL and ISML as *L*_2_. The molecular composition (or content) of WL and ML in the three groups of uninterrupted intervals (min–Mw–max; min–4000–10,000–max; min–2000–4000–6000–8000–10,000–20,000–50,000–max) were calculated according to Eq. (8-1). Similarly, the molecular compositions (or contents) of the fractions (SWL, ISWL, SML, and ISML) were calculated using Eq. (8-2). The molecules smaller and larger than Mw (5653 for WL and 6553 for ML) were defined as the small and large molecules, respectively. WL contained 30.1% small molecules and 69.9% large molecules under two intervals (Tables S1, S2 and Fig. 2a). After fractionation, the contents of the small molecules were increased to 55.3% for SWL and decreased to 10.1% for ISWL (Fig. 2a). The contents of the large molecules were decreased to 45.7% for SWL and increased to 89.9% for ISWL (Fig. 2a). The molecular compositions of parent ML, SML, and ISML were also quantified: the contents of the small molecules of the fractionated SML and ISML were increased to 59.9% and decreased to 13.1%, respectively, from 30.7% of ML (Fig. 2b). The large molecules of the fractionated SML and ISML were decreased to 40.1% and increased to 86.9%, respectively, from 69.3% of ML (Fig. 2b). Based on the established integral method, the fractionation trend of small and large molecules was quantitatively analyzed.

**Figure 3:**
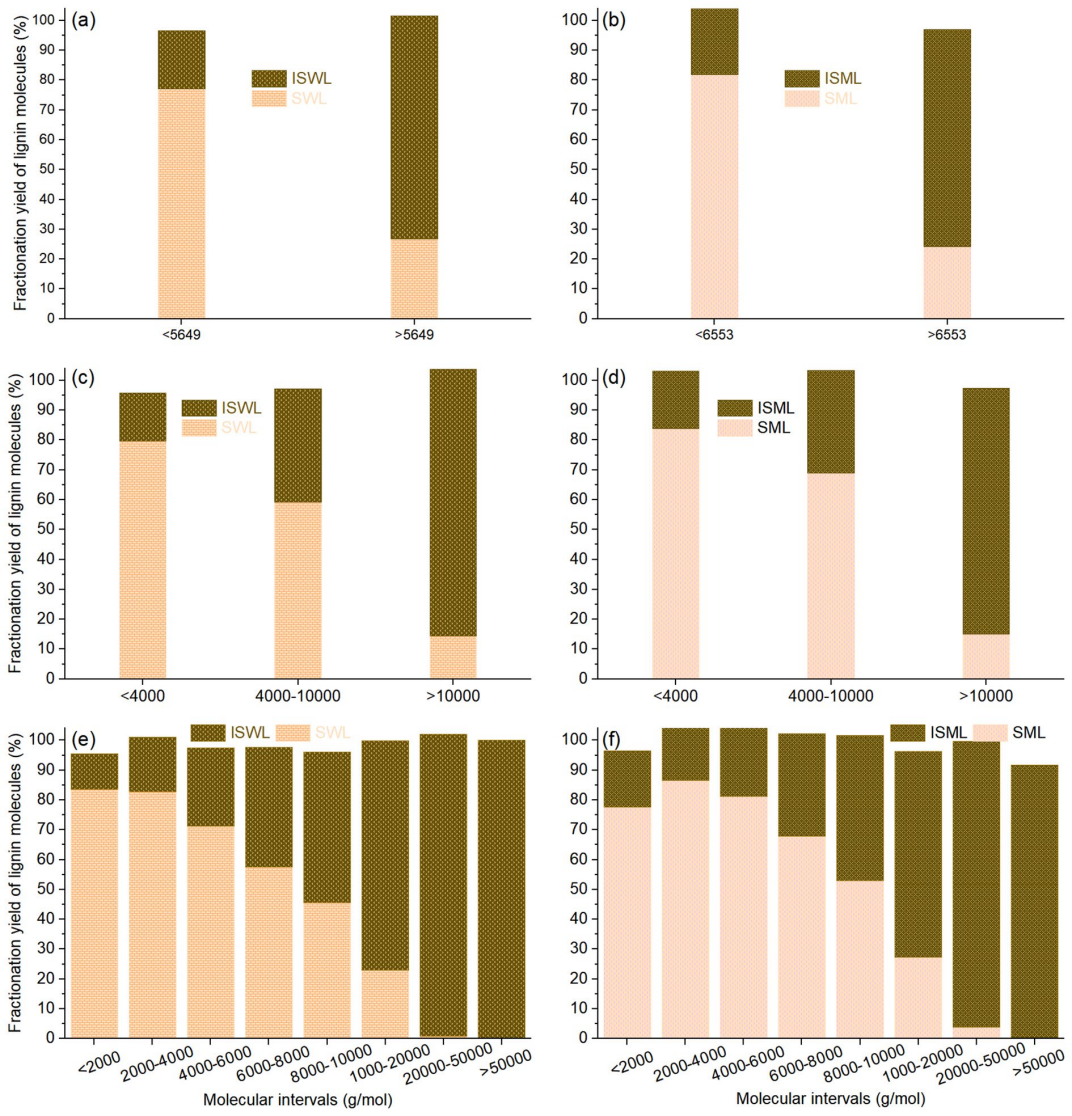
Fractionation yields of molecules belonging to different molecular intervals: SWL and ISWL under (**a**) 2, (**c**) 3, and (**e**) 8 intervals; SML and ISML under (**b**) 2, (**d**) 3, and (**f**) 8 intervals.

**Figure 4:**
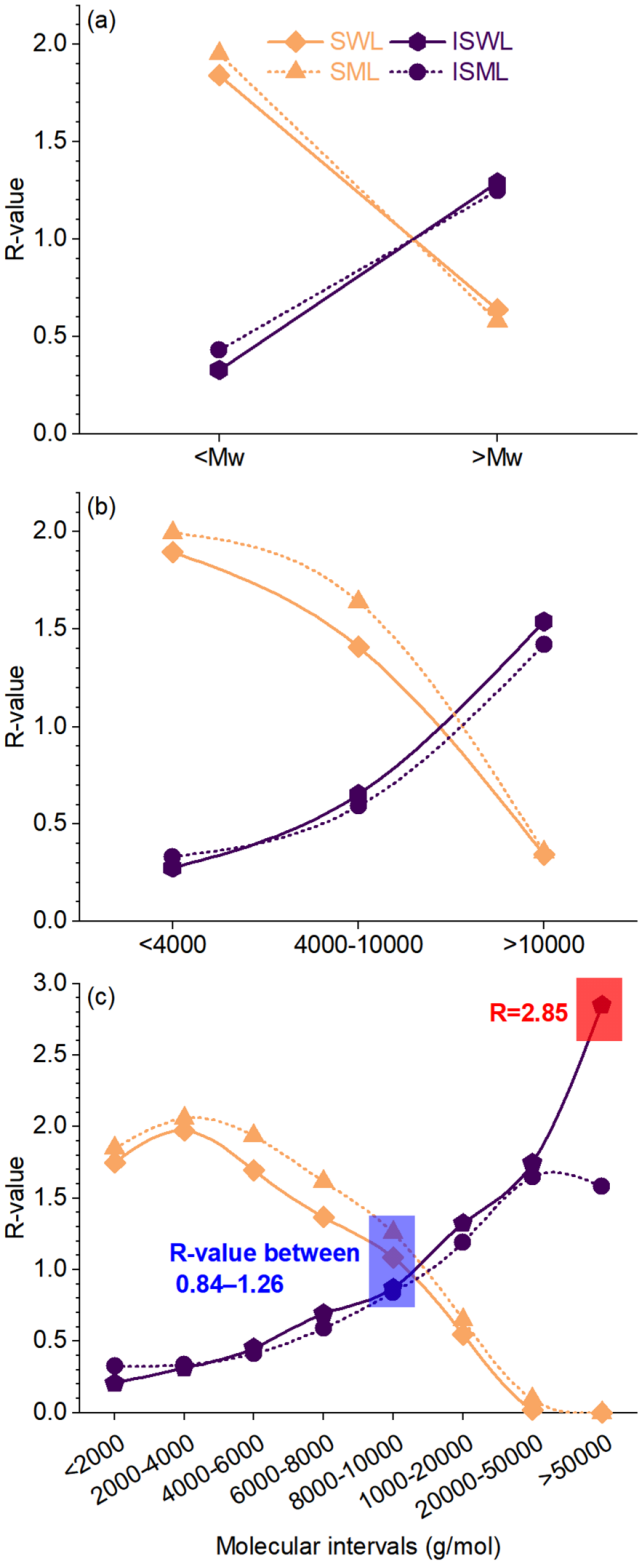
Ratio (R) between the yields of the fractions belonging to a certain molecular interval and the yield of fraction: R under (**a**) 2, (**b**) 3, and (**c**) 8 intervals; Mw was 5653 for WL and 6553 for ML.

The yield of the fraction belonging to a certain molecular interval was quantitatively calculated using Eq. (10), and the results are shown in Table S3 and Fig. 3. The yields of the small and large molecules for SWL were 77.1% and 26.9%, respectively, based on the corresponding small and large molecules of WL (Fig. 3a). The ratios between the yields of the small and large molecules in SWL and the yield of SWL ($R_{{SWL \to \min { - }Mw}}$ and $R_{{SWL \to Mw{\text{ - max}}}}$) were calculated according to Eq. (12). If the small and large molecules of WL were randomly fractionated into SWL, the $R_{{SWL \to \min { - }Mw}}$ and $R_{{SWL \to Mw{\text{ - max}}}}$ should be 1.0. However, the result showed that the values of $R_{{SWL \to \min { - }Mw}}$ and $R_{{SWL \to Mw{\text{ - max}}}}$ were 1.84 and 0.64, respectively (Table S4 and Fig. 4a). This indicates that 84% of the small molecules were fractionated into SWL because they were easily soluble, while 36% of large molecules were not fractionated into SWL as they were difficult to dissolve. In contrast, the yields of the small and large molecules for ISWL were 19.4% and 74.6%, respectively, based on the corresponding molecules of WL (Fig. 3a). The ratios between the yields of the small and large molecules of ISWL and the yields of ISWL, $R_{{ISWL \to \min { - }Mw}}$, and $R_{{ISWL \to Mw{\text{ - Max}}}}$ were 0.33 and 1.29, respectively (Table S4 and Fig. 4a). This indicates that 67% of the small molecules could not be fractionated into ISWL as they could be easily solubilized in solvents, while 29% of the large molecules were fractionated into ISWL, as it was difficult to dissolve them into solvents. The ratios between the yields of the small and large molecules and the yield of SML ($R_{{SML \to \min { - }Mw}}$ and $R_{{SML \to Mw{\text{ - max}}}}$) were 1.95 and 0.58, respectively (Fig. 4a). The ratios corresponding to the yield of ISML ($R_{{ISML \to \min { - }Mw}}$ and $R_{{ISML \to Mw{\text{ - max}}}}$) were 0.43 and 1.25, respectively (Fig. 4a). The easier dissolution of the small molecules was validated^23,24^. More importantly, this is the first time that a quantitative method to calculate the fractionation yield of the lignin molecules has been proposed.

More than 10% difference in the molecule contents of the parent lignin and their fractions was observed under 3 intervals (Fig. 2c and d). For example, the molecule contents corresponding to WL, SWL, and ISWL were 19.6%, 37.3%, and 5.4% under the interval of min–4000 g/mol, respectively; 33.0%, 46.5%, and 21.6% under the interval of 4000–10,000 g/mol, respectively; and 47.4%, 16.3%, and 73.0% g/mol under the interval of 10,000–max g/mol, respectively (Fig. 2c). Under eight intervals, WL, SWL, and ISWL showed similar molecule contents of 9.40%, 10.2%, and 8.20%, respectively, under the interval of 8000–10,000 g/mol (Fig. 2e). This indicates that WL belonging to the interval of 8000–10,000 g/mol has little influence on fractionation. This conclusion was reached when the molecules were divided into eight intervals instead of two or three. This fully reflects the advantage of the integral method. This conclusion cannot be drawn by studying the differences in the Mn, Mw, and PDI values recorded for the parent lignin molecules and the fractions. Interestingly, the contents of ML, SML, and ISML were similar and in the range of 6.62–9.92% under the interval of 8000–10,000 g/mol (Fig. 2f). Therefore, molecules of ML in the interval of 8000–10,000 g/mol have little effect on fractionation. This observation is similar to the observation made for WL. In particular, the R-values of SWL, ISWL, SML, and ISML at the interval of 8000–10,000 ($R_{SWL \to 8000 - 10000}$, $R_{ISWL \to 8000 - 10000}$, $R_{SML \to 8000 - 10000}$, and $R_{ISML \to 8000 - 10000}$) were 1.09, 0.87, 1.26, and 0.84 (Fig. 4b and c), respectively. All $R_{SWL \to 8000 - 10000}$, $R_{ISWL \to 8000 - 10000}$, $R_{SML \to 8000 - 10000}$, and $R_{ISML \to 8000 - 10000}$ were close to 1.0, indicating that the molecules between 8000–10,000 g/mol have little effect on the process of fractionation. The results agree well with the results obtained by studying the molecular composition under eight intervals. Other lignin samples can potentially exhibit different molecular fractionation trends between the interval of 8000–10,000 g/mol for WL and ML.

Under conditions of three intervals, the yields of the fraction under the molecular interval of min–4000, 4000–10,000, and 10,000–max were determined and compared with the yields of the molecules of WL in the same interval: (1) a decrease in the yield was observed for SWL (79.7%, 59.2%, and 14.4%), and (2) an increase in the yields equal to 16.1%, 37.9%, and 89.4% was recorded for ISWL (Fig. 3c). The smaller the molecules, the higher the solubility, and the easier it was to fractionate the molecules into the soluble fraction (Fig. 3c and d). The results could be easily arrived at when the molecules were divided into 8 intervals (Fig. 3e and f). With the increase in the molecular interval from min–2000 to 50,000–max, the yields of the molecules decreased from 83.5% to 0 for SWL and increased from 11.9 to 100% for ISWL (Fig. 3e). It was also observed that the R-value decreased from 1.75 to 0 for SWL and increased from 0.21 to 2.85 for ISWL (Fig. 4b and c).

### Data accuracy

The total yield of soluble and insoluble fractions belonging to certain molecular intervals should be equal to 100%. For example, the total yield of SWL and ISWL in the molecular interval of 8000–10,000 g/mol was 96.2% (Fig. 3e), and the total yield of SML and ISML in this interval was 101.6% (Fig. 3f). The total yield of the fractions belonging to all intervals (except the molecular interval of 50,000–max) was in the range of 95.4–106.7% (Fig. 3). This indicates that the error was within 7.0%. The total yields of the fractions belonging to the molecular interval of 50,000–max for WL and ML were 165.1% and 91.7% (Fig. 3), respectively, which were significantly lower than 100%. WL and ML showed lower molecule content of 2.3% and 4.7%, respectively, in the interval of 50,000–max (Fig. 2e and f). A small absolute error can potentially result in the above-mentioned large relative errors.

Considering the fact that the yields of the soluble (SWL and SML) and insoluble (ISWL and ISML) fractions were 42 ± 1% and 58 ± 1%, the maximum R-value for the soluble and insoluble fractions should be 2.38 (= 1/0.42) and 1.72 (= 1/0.58), respectively. Only the $R_{ISWL \to 50000 - \max }$ value of 2.85 (Fig. 3e) far exceeded the maximum value of 2.38. This was potentially induced by the lower molecule content (2.3%) of WL in this interval.

## Conclusion

In this study, a simple integral method for quantitative calculation the molecular weight characteristics of lignin based on molecular weight distribution curve (Mw–Mw%) was proposed. The accuracy of this method has also been analyzed and proved. The fractionation tendency of two lignin samples at certain molecular intervals were clearly revealed based on this method. The molecules in the range of 8000–10,000 g/mol for WL and ML showed little influence on the process of fractionation, whereas the molecules whose weights were below 8000 g/mol were more likely to be fractionated into soluble fractions. The molecules whose weights were higher than 10,000 g/mol are more likely to be fractionated into insoluble fractions. The established integral method is a key tool for understanding the controllable fractionation and high value-added utilization of heterogeneous lignin. More interesting results are likely to be obtained based on the integral method after the lignin is fractionated into more than 2 fractions.

## Supplementary Information

Supplementary Information.

## Data Availability

Supplementary data have been submitted. The datasets used and/or analysed during the current study available from the corresponding author on reasonable request.
