# Supplementary material for: An integral method for determining the molecular composition of lignin and its application
Source: Sci Rep. 2022 Nov 9;12:19136. doi: 10.1038/s41598-022-23884-5 (PMC9646882; doi:10.1038/s41598-022-23884-5)
Supplement: Supplementary file 1 — Supplementary Information. [file 41598_2022_23884_MOESM1_ESM.pdf]

Supplementary Information for

An Integral Method for Determining the Molecular Composition of Lignin and its Application

Qingzhi Ma[a] *, Xuejin Zhang[a] *

[a] Key Laboratory of recycling and Eco-treatment of Waste Biomass of Zhejiang Province, School of Environmental and Nature Resources, Zhejiang University of Science and Technology, Hangzhou, Zhejiang 310023, China

*Corresponding author: maqingzhi@zust.edu.cn; xuejinzhang@126.com

Table S1 Molecule content calculation process (SWL, WL, and ISWL were shown as an example)

Molecular interval (g/mol)	Integral result based on Mw-Mw%			Molecule content (%)		
	SWL[a]	WL[b]	ISWL[a]	SWL[c]	WL[d]	ISWL[c]
min-max	761788	1492401	3268619	100	100	100
min–2000	87882	98483	44278	11.5	6.60	1.35
2000–4000	195923	194632	133337	25.7	13.0	4.08
4000–6000	158636	183190	181464	20.8	12.3	5.55
6000–8000	117480	168166	254966	15.4	11.3	7.80
8000–10000	77910	140467	267925	10.2	9.40	8.20
10000–20000	121116	433228	1256346	15.9	29.0	38.4
20000–50000	2842	240356	919078	0.40	16.1	28.1
50000–max	0	33878	211226	0	2.3	6.46

a) the integra results of total and certain interval of fractions are calculated by equation of

$$\text{integral of total (or certain interval)} = \int_{\min}^{\max} Mw_x \% dMw_x$$;

b) the integra results of total and certain interval of WL are calculated by equation of

$$\text{integral of total (or certain interval)} = \int_{\min}^{\max} Mw_0 \% dMw_0$$;

c) calculated based on Eq. (8-2) $m_{x\to a-b}(\%) = \frac{\int_a^b Mw_x \% \times dMw_x}{\int_{\min}^{\max} Mw_x \% \times dMw_x}$;

d) calculated based on Eq. (8-1) $m_{0\to a-b}(\%) = \frac{\int_a^b Mw_0 \% \times dMw_0}{\int_{\min}^{\max} Mw_0 \% \times dMw_0}$.

Table S2 Molecular composition of lignin and their fractions under different molecular intervals

Molecular intervals (g/mol)		Molecule content in certain molecular interval (%)					
		SWL	WL	ISWL	SML	ML	ISML
two intervals	< Mw[a]	55.3	30.1	10.1	59.9	30.7	13.1
	> Mw[a]	45.7	69.9	89.9	40.1	69.3	86.9
three intervals	min–4000	37.3	19.6	5.4	33.8	17.0	5.6
	4000–10000	46.5	33.0	21.6	46.8	28.5	16.9
	10000–max	16.3	47.4	73.0	19.5	54.5	77.5
eight intervals	min–2000	11.5	6.60	1.35	9.73	5.27	1.71
	2000–4000	25.7	13.0	4.08	24.0	11.7	3.92
	4000–6000	20.8	12.3	5.55	21.2	11.0	4.56
	6000–8000	15.4	11.3	7.80	15.6	9.65	5.73
	8000–10000	10.2	9.40	8.20	9.92	7.88	6.62
	10000–20000	15.9	29.0	38.4	17.3	26.5	31.6
	20000–50000	0.40	16.1	28.1	2.17	23.3	38.5
	50000–max	0	2.3	6.46	0	4.7	7.43

a, Mw was 5653 and 6553 g/mol for WL and ML, respectively.

Table S3 Fractionation yield of lignin molecules in different molecular intervals

Molecular intervals (g/mol)		Molecule yield in certain molecular interval (%)					
		SWL[b)]	ISWL[b)]	Sum of SWL and ISWL	SML[b)]	ISML[b)]	Sum of SML and ISML
two intervals	< Mw[a)]	77.1	19.4	96.5	81.9	24.8	106.7
	> Mw[a)]	26.9	74.6	101.5	24.3	72.7	97.0
three intervals	min–4000	79.7	16.1	95.7	83.8	19.3	103.0
	4000–10000	59.2	37.9	97.2	68.9	34.4	103.3
	10000–max	14.4	89.4	103.8	15.0	82.4	97.4
eight intervals	min–2000	83.5	11.9	95.4	77.6	18.8	96.4
	2000–4000	82.8	18.1	101.0	86.5	19.5	106.0
	4000–6000	71.3	26.2	97.5	81.2	24.1	105.3
	6000–8000	57.5	40.2	97.6	67.9	34.4	102.3
	8000–10000	45.6	50.5	96.1	52.9	48.7	101.6
	10000–20000	23.0	76.8	99.8	27.3	69.0	96.3
	20000–50000	0.9	101.3	102.1	3.9	95.8	99.7
	50000–max	0	165.1	165.1	0	91.7	91.7

a) Mw was 5653 and 6553 g/mol for WL and ML, respectively; b) the yield of SWL and ISML was both 42 ± 1.0%, and the yield of ISWL and ISML was both 58 ± 1.0%.

Table S4 The ratio of fraction yield in certain molecular intervals and fraction yield (R)

Molecular intervals (g/mol)		R			
		SWL[b)]	ISWL[b)]	SML[b)]	ISML[b)]
two intervals	< Mw[a)]	1.84	0.33	1.95	0.43
	> Mw[a)]	0.64	1.29	0.58	1.25
three intervals	min–4000	1.90	0.28	1.99	0.33
	4000-10000	1.41	0.65	1.64	0.59
	10000–max	0.34	1.54	0.36	1.42
eight intervals	min–2000	1.75	0.21	1.85	0.32
	2000–4000	1.97	0.31	2.06	0.34
	4000–6000	1.70	0.45	1.93	0.41
	6000–8000	1.37	0.69	1.62	0.59
	8000–10000	1.09	0.87	1.26	0.84
	10000–20000	0.55	1.32	0.65	1.19
	20000–50000	0.02	1.75	0.09	1.65
	50000–max	0	2.85	0	1.58

a) Mw was 5653 and 6553 g/mol for WL and ML, respectively; b) the yield of SWL and ISML was both 42 ± 1.0%, and the yield of ISWL and ISML was both 58 ± 1.0%.
